# Transfobia no Brasil em contexto pandêmico: percepções e ações de coletivos políticos que atuam em prol da população trans

**DOI:** 10.1590/0102-311XPT161424

**Published:** 2025-11-10

**Authors:** Aline Maia Diniz, Vera Lucia Marques da Silva

**Affiliations:** 1 Escola Nacional de Saúde Pública Sergio Arouca, Fundação Oswaldo Cruz, Rio de Janeiro, Brasil.

**Keywords:** Saúde das Minorias, COVID-19, Travestilidade, Transexualidade, Política Pública, Minority Health, COVID-19, Transvestism, Transsexualism, Public Policy, Salud de la Minorias, COVID-19, Travestismo, Transexualidad, Política Pública

## Abstract

O contexto da pandemia de COVID-19 no Brasil foi marcado por uma série de medidas para contenção do vírus, que tiveram repercussões específicas sobre os diferentes grupos sociais, especialmente os mais vulnerabilizados, como transexuais e travestis. O presente artigo objetiva compreender particularidades da transfobia no Brasil no contexto da pandemia de COVID-19 e a atuação de organizações e coletivos políticos diante desse cenário, levando em conta o ineditismo e a relevância de sua contribuição, dada a escassez de estudos voltados à compreensão do fenômeno transfóbico em contextos de grave crise social. Adotando uma abordagem qualitativa, foram realizadas cinco entrevistas com representantes de coletivos trans de abrangência local, regional e nacional. O tratamento do material recolhido empregou a técnica de análise de conteúdo, conforme preconizada por Bardin, e privilegiou aportes teóricos *queer*. A organização dos resultados se deu em dois eixos temáticos: (1) transfobia em relações cotidianas; e (2) desafios no acesso a políticas públicas. Este eixo foi subdividido em: (a) política de educação e mercado de trabalho; (b) política de assistência social e segurança alimentar; e (c) política de saúde e saúde mental. O estudo aponta que, na avaliação dos coletivos entrevistados, a pandemia intensificou uma série de desigualdades e violências que já eram experienciadas por travestis e transexuais, indicando o relevo da in/ação do Estado, ele mesmo agente de transfobia.

## Abstract (en)

The COVID-19 pandemic in Brazil was marked by a set of measures to contain the virus, which had specific repercussions in different social groups, especially the most vulnerable, such as transvestites and transgender individuals. The aim of the present article was to investigate the particularities of transphobia in Brazil in the context of the COVID-19 pandemic as well as the actions of organizations and political groups in this scenario. The novelty and relevance of this study is evident by the scarcity of investigations focused on understanding the transphobic phenomenon in the context of a serious social crisis. Adopting a qualitative approach, five interviews were conducted with representatives of trans support groups on the local, regional, and national levels. The treatment of the material collected involved the content analysis technique proposed by Bardin and prioritized queer theoretical contributions. The results were organized into two major topics: (1) transphobia in everyday relationships; and (2) challenges in accessing public policies, the latter of which was subdivided: (a) education policy and the job market; (b) social assistance policy and food security; and (c) health policy and mental health. In the assessment of the groups interviewed, the pandemic intensified a series of inequalities and violence already experienced by transvestites and transgender individuals, highlighting the (in)action of the government, which itself is an agent of transphobia.

## Abstract (es)

El contexto de la pandemia de la COVID-19 en Brasil ha sido marcado por una serie de medidas para evitar la propagación del virus, las cuales tuvieron repercusiones específicas en diferentes grupos sociales, especialmente entre los más vulnerables, como los transexuales y travestis. Este artículo tiene como objetivo identificar las particularidades de la transfobia en Brasil en el contexto de la pandemia de la COVID-19 y el papel de las organizaciones políticas y colectivos en este escenario, lo que demuestra aportes valiosos y con originalidad dada la escasez de estudios para comprender el fenómeno transfóbico en contextos de grave crisis social. A partir de un enfoque cualitativo, se realizaron cinco entrevistas con representantes de colectivos trans de alcance local, regional y nacional. Para el procesamiento del material recolectado se utilizó la técnica de análisis de contenido de Bardin, y se centró en las contribuciones teóricas queer. Los resultados se organizaron en dos ejes temáticos: (1) transfobia en las relaciones cotidianas; y (2) desafíos en el acceso a las políticas públicas. Este último eje se subdividió en (a) política de educación y mercado laboral; (b) política de asistencia social e seguridad alimentaria; y (c) política de salud y salud mental. Este estudio señala que, en la evaluación de los colectivos entrevistados, la pandemia intensificó una serie de desigualdades y violencias que ya venían experimentando los travestis y transexuales, y esto revela la relevancia de la in/acción del Estado, el propio agente de transfobia.

## Apresentação

Frente à escassez de dados oficiais sobre transfobia, organizações da sociedade civil realizam mapeamentos, objetivando visibilizar o fenômeno. É o caso da Associação Nacional de Travestis e Transexuais (ANTRA), que indica que, a cada 48 horas, uma pessoa trans, englobando travestis e transexuais, é assassinada no Brasil e a cada 10 desses homicídios no mundo, quatro ocorrem aqui. O país é considerado o que mais mata pessoas deste grupo social no mundo e onde sua expectativa de vida é de 35 anos (metade da média nacional). Além de violência física, outros tipos acometem esta população diariamente, por vezes de forma velada, o que dificulta a identificação, a notificação e o enfrentamento. Excluída das políticas públicas, do acesso à educação e ao mercado de trabalho formal e, em muitos casos, de suas próprias famílias, parte significativa dessa população vive em situação de extrema vulnerabilidade, intensificada após a eleição de um Governo Federal explicitamente transfóbico (2019-2022) ^1^.

O vírus da COVID-19 chegou ao Brasil em dezembro de 2019, o que requereu medidas rígidas como isolamento social, fechamento de diversos setores econômicos e direcionamento do atendimento dos serviços de saúde aos casos da doença e alguns outros considerados essenciais. Foi em 2020 que o número de assassinatos de pessoas trans ocorreu em maior número: 175 homicídios foram registrados frente a 124 no ano anterior, o que foi relacionado ao fato de que 70% desta população não conseguiu acesso às políticas emergenciais do Estado ^1^. Por falta de alternativas, continuaram trabalhando nas ruas, como profissionais do sexo, o que representou maior exposição ao vírus e à violência. Estima-se que, em 2017, cerca de 90% de travestis e mulheres transexuais exerciam essa atividade, dada a falta de oportunidades no mercado de trabalho ^2^. Neste cenário, a atuação de coletivos trans foi crucial, promovendo desde distribuição de cestas básicas até apoio emocional, como veremos adiante.

O ativismo trans no Brasil tem longa história, entrelaçada à luta de outras identidades que hoje compõem o Movimento LGBTI+ ^3^, ao enfrentamento à epidemia de aids e à violência policial. Data de 1995 o VIII Encontro Brasileiro de Gays e Lésbicas que reconheceu, após embates, travestis como sujeitos políticos do movimento e fundou a Associação Brasileira de Gays, Lésbicas e Travestis (ABGLT). Essa entrada “institucionalizada” das travestis no movimento gay e lésbico foi consequência da mobilização política da categoria, que já havia realizado dois encontros promovidos pela então Associação de Travestis e Liberados (Astral) ^4^. Esta foi a primeira organização ativista de travestis da América Latina e a segunda do mundo, se constituindo em resposta à violência policial em áreas de atuação de profissionais do sexo na cidade do Rio de Janeiro. A constituição da categoria “transexual” como distinta da de “travesti” data do fim dos anos 1990 e início dos anos 2000. Após uma série de mudanças em sua nomenclatura, a Astral se torna, no início deste século, ANTRA, incorporando a categoria “transexuais” ^5^. Em 2005, no I Congresso realizado pela ABGLT, estabeleceu-se o uso dos termos gay, lésbica, bissexual e transgênero nos documentos oficiais. A adoção do termo transgênero revela influência internacional sobre o ativismo brasileiro e acirra as tensões identitárias, seja pelo desconhecimento do termo no país à época, seja por aglutinar identidades distintas, invisibilizando-as ^4^ ^,^ ^5^.

O presente artigo objetiva compreender particularidades da transfobia no Brasil no contexto da pandemia de COVID-19 e a atuação de coletivos políticos frente a esse cenário. Por transfobia compreendemos o preconceito contra pessoas transexuais ou travestis e que envolve diversos tipos de violências ^6^. Optamos pelo uso do termo “coletivo político” como englobante de organizações formais e informais que possuem articulação política, atuando como movimento social e direcionando suas ações em prol da defesa de direitos de grupos sociais específicos. O ineditismo deste estudo consiste em apresentar, a partir da avaliação de representantes de coletivos políticos, implicações entre desigualdades e violências experienciadas por esta população e o enfrentamento à pandemia de COVID-19. Sua relevância se dá frente à significativa exclusão social vivenciada por pessoas trans e à escassez de estudos a respeito em graves crises sociais, como as sanitárias, particularmente, se compararmos ao acúmulo de pesquisas sobre a acentuação da violência de gênero contra mulheres cis nestes mesmos cenários.

## Métodos

Este estudo adota abordagem qualitativa, privilegiando aportes teóricos *queer*. A partir da década de 1980, *queer* passou a denominar uma linha teórico-metodológica e política, que ganhou espaço entre os estudos de gênero. Articulando filosofia, estudos culturais norte-americanos e pós-estruturalismo francês, problematiza concepções clássicas de sujeito, identidade e agência ^7^. É aqui representada por autoras como Butler ^8^, Bento et al. ^9^ e Nascimento ^10^.

O desenho metodológico incluiu a realização de entrevistas, já que podem fornecer dados importantes sobre o cenário social, auxiliando na compreensão das relações entre processos e fenômenos sociais ^11^ ^,^ ^12^. Para identificação de potenciais participantes de pesquisa, consultamos documentos das Conferências Nacionais voltadas aos direitos LGBT por pressupormos que lá estavam representados coletivos de maior reconhecimento nacional. Elegemos como critérios de seleção: tempo de existência do grupo (no mínimo, cinco anos); atuação em defesa dos direitos trans; atividade em qualquer região brasileira e durante a pandemia de COVID-19. A busca pelos contatos institucionais dos 13 selecionados se deu por meio de suas páginas digitais, o que permitiu envio de convite por correio eletrônico ou redes sociais. Destes, apenas dois responderam, recusando-se a participar. A ida em eventos que envolveram lideranças trans, contudo, demonstrou ser caminho profícuo para novos contatos, especialmente o Encontro Nacional da ANTRA, realizado em agosto de 2022 na cidade de Niterói (Rio de Janeiro).

Ao fim, foram realizadas cinco entrevistas de uma hora de duração, em média, sendo uma presencial e quatro remotas; todas guiadas por roteiro semiestruturado. Dada sua natureza dialogal, este possibilita maior flexibilidade para abordagem de aspectos de interesse do estudo ^11^. Os tópicos elencados versavam sobre o perfil dos coletivos, sua atuação durante a pandemia, dificuldades enfrentadas por trans e relação entre movimento social e Estado naquele contexto. Não houve teste piloto do roteiro, visto que sua função era a de orientar a conversa ^11^. Mesmo assim, após a realização da primeira, o consideramos adequado. As entrevistas foram conduzidas pela primeira autora, em agosto de 2022, que as gravou e as transcreveu. Esta já havia realizado pesquisas qualitativas, inclusive nesta temática. Sua experiência profissional em um serviço do processo transexualizador contribuiu positivamente para o desenvolvimento do trabalho.

Participaram desta investigação representantes dos coletivos: Instituto Trans da Maré, Grupo Pela Vidda (GPV), Liga Transmasculina João W. Nery, Fórum Nacional de Travestis e Transexuais Negras e Negros (FONTRANS) e Instituto Brasileiro de Transmasculinidades (IBRAT). Considerando o foco de atuação destes grupos, percebe-se sua diversidade: mulheres trans e travestis, homens trans e pessoas transmasculinas, população LGBTI+ com HIV, população trans negra. Quanto aos níveis de atuação, tem-se local, regional e nacional. A organização mais antiga é o GPV, que iniciou suas atividades em 1989; os demais se constituíram a partir de 2012. No Quadro 1 é possível conhecer o perfil dos participantes:

**Quadro 1:** Perfil dos coletivos.

ORGANIZAÇÃO/ COLETIVO	ANO DE FUNDAÇÃO	ABRANGÊNCIA GEOGRÁFICA	POPULAÇÃO ATENDIDA
Instituto Trans da Maré	Não soube informar ao certo, mas confirmou ter mais de 5 anos	Local	Mulheres trans
Liga Transmasculina João W. Nery	Formalizado em 2019, mas atua desde 2013	Nacional	Pessoas transmasculinas e homens trans
Grupo Pela Vidda	1989	Regional/Nacional	População LGBTI+ e pessoas vivendo com HIV, mas com projetos prioritários para pessoas trans
Fórum Nacional de Travestis e Transsexuais Negras e Negros	2014	Nacional	Travestis e transexuais negras e negros
Instituto Brasileiro de Transmasculinidades	2012	Nacional	Pessoas transmasculinas

Fonte: elaboração própria.

As entrevistas foram examinadas por meio da técnica de análise de conteúdo, desenvolvida em três etapas: (1) descrição: enumeração das características do texto de forma resumida; (2) inferência: dedução das relações entre uma proposição oriunda da descrição com outras já consideradas verdadeiras; (3) interpretação: busca pela significação concedida a estas características. O tratamento dado, portanto, consiste em leituras sistemáticas, busca por palavras-chaves para identificar o que pode ser posto em evidência e interpretação ^13^.

A pesquisa foi aprovada pelo Comitê de Ética em Pesquisa da Escola Nacional de Saúde Pública Sergio Arouca, Fundação Oswaldo Cruz (CAAE 58094122.8.0000.5240). As pessoas entrevistadas foram informadas acerca da proposta do estudo, do desejo de ouvir representantes de coletivos, dos riscos envolvidos e das estratégias para minimizá-los. Ao serem questionadas sobre se concordavam ou não com a divulgação do nome do coletivo, todas autorizaram. Todavia, omitimos os nomes pessoais, optando por referendar a partir da identificação do coletivo.

## Resultados e discussão

A análise das entrevistas possibilitou identificar dois eixos temáticos: (1) transfobia em relações cotidianas; e (2) desafios no acesso a políticas públicas. Este eixo foi subdividido em: (a) política de educação e mercado de trabalho; (b) política de assistência social e segurança alimentar; e (c) política de saúde e saúde mental.

### Transfobia em relações cotidianas

Quando questionadas/os sobre se percebiam recrudescimento da transfobia durante o contexto pandêmico, obtivemos unanimidade no assentimento, tanto no ambiente doméstico, quanto no digital e nas ruas. A interseccionalidade entre transgeneridade, raça e classe foi ressaltada para denunciar o agravamento das violências sofridas por pessoas trans, quando negras e residentes em favelas. A interseccionalidade é uma conceituação do problema que busca capturar as consequências estruturais e dinâmicas da interação entre sistemas de subordinação, como o racismo, o patriarcalismo e a opressão de classe, que re/produzem desigualdades estruturais ^14^. Nesse sentido, destacamos o seguinte relato:

“*A pessoa trans branca é vista na sociedade de uma forma diferente, principalmente se ela tiver um poder aquisitivo, se ela for de classe média, classe média alta. Então, ela é tratada de uma forma diferente. Não que ela não sofra violência, mas a violência não é tão brutal, ela não é tão nítida quanto a violência que pessoas trans* [de camadas populares], *principalmente as travestis negras, sofrem. E mesmo que essa travesti preta tenha uma condição financeira boa, que esteja na classe média, que trabalhe, que tenha um curso, doutorado, mestrado, o tratamento pra ela é sempre diferente. Tem aquela questão, ela vai ser objetificada porque é uma travesti, é um corpo preto. Então, não tem direitos, né? Então, ela pode ser assediada, ela pode ser violentada de todas as formas, ela pode ser inclusive morta*” (FONATRANS).

Nos casos de transfeminicídio, o crime não costuma provocar a mesma indignação que ocorre quando há morte de mulheres cis ^15^, uma vez que, por um lado, está associado a uma espécie de assepsia da humanidade e, por outro, cumpre uma função social: os corpos desfigurados, que frequentemente caracterizam esse tipo de homicídio, contribuem para manutenção e reprodução da cisgeneridade, que impõe a genitália como definidora da identidade de gênero. Essa pretensa assepsia decorre da recusa em reconhecer como humanas as vidas trans ^10^. Neste caso, se certas vidas não são reconhecidas como vidas, haja visto determinados enquadramentos epistemológicos, elas jamais serão vividas ou perdidas no sentido pleno desses termos ^16^.

A violência sexual e seu silenciamento contra homens trans/transmasculinos em territórios dominados por grupos armados também foi apontada:

“*Eu vejo muito menino sendo estuprado, muito, muitos relatos, assim, e assim, estuprado dentro de favela, a maioria deles. Aí, eu vou fazer o quê?* [pausa, silêncio] (...) *por miliciano, alguns,* [silêncio] *saca? É isso, assim, então a gente tem que ficar criando estratégias, recolhendo tudo, pra quando denunciar, ainda a gente tem risco ainda, mas a gente precisa pensar nessas estratégias de sobrevivência*” (Liga Transmasculina João W. Nery).

Nesse caso, a transfobia é atravessada pela violência territorial, a ponto de ser cometida também por agentes que deveriam oferecer segurança, o que dificulta ações de enfrentamento e mesmo a denúncia:

“*Vai falar para quem? Pois é! E a gente também não pode levar uma ideia de que a favela é lugar de perigo, porque não é. É uma outra sociedade que faz com que a favela seja lugar de perigo.* (...) *Aí, como que a gente vai denunciar? Eu sou só um homem preto*” (Liga Transmasculina João W. Nery).

O 1º Dossiê Anual do Observatório de Violências LGBTI+ em favelas ^17^, que ouviu 1.705 pessoas LGBTI+ moradoras de favelas do estado do Rio de Janeiro, reforça nossos achados de pesquisa: 47,8% dos respondentes já tiveram suas casas invadidas durante operações policiais. Entre a população negra, este percentual chegou a 39,6%. Cinquenta e sete vírgula nove por cento foram abordados pela polícia, antes e depois dos 18 anos de idade. Nas abordagens, 24,3% se sentiram ameaçadas por sua identidade de gênero e/ou orientação sexual, sendo 37,7% destas pessoas LGBTI+ negras. Entre trans e não-bináries (expressão em linguagem agênera), 24,69% foram vítimas de ameaças. Com isso, é possível inferir o peso da interseccionalidade entre gênero, raça e classe sobre a população trans e sinaliza a complexidade da realidade social.

### Desafios no acesso a políticas públicas

A dificuldade de acesso às políticas públicas é um fator que exerce efeito negativo considerável na rotina de pessoas trans, apontando transfobia institucional, ou seja, formas institucionalizadas de discriminação, criminalização, patologização e estigmatização destas ^18^. Inúmeros foram os relatos sobre dificuldades de acesso a hospitais e escolas, por exemplo. Optamos por analisá-los a partir de três subeixos, como já mencionamos.

#### Política de educação e mercado de trabalho

Apesar da política de educação ser considerada acessível, a permanência de discentes trans, frequentemente, é impossibilitada pela transfobia, que gera insegurança e exclusão. Vale ressaltar que a escola, ao reiterar normativas patriarcais de gênero, inclusive por meio da vigilância de corpos e comportamentos, potencializa situações de discriminação. Aqueles que não se enquadram nas normativas estão sujeitos a sanções imputadas pela escola e por colegas de classe ^9^.

Algumas das situações compartilhadas se relacionam à impossibilidade de uso de banheiro condizente à identidade de gênero e do nome social, a despeito de decreto que regulamenta o uso do nome social no âmbito da administração pública federal, reconhecendo a identidade transgênera ^19^.

“*Tem universidades e escolas hoje que não sabem nem o que é o nome social ainda, uma política que nasceu em 2016. Eu mesmo entrei no doutorado, tenho um ano e meio* (...) *e eles não sabiam o que era a política de nome social*” (IBRAT).

Representante do GPV compartilhou resultados de pesquisa para conhecer o perfil das pessoas atendidas pelo grupo. Um dos dados indica que homens trans têm mais oportunidades de estudo e trabalho que mulheres trans: “*a gente descobriu também que os homens trans têm uma escolaridade maior e conseguem trabalho mais fácil do que as mulheres*”, o que aponta para um dos efeitos da passabilidade social dos primeiros. Esta ocorre quando a produção de características corporais e a adoção de atributos de comportamento congruentes com a identidade de gênero assumida é socialmente inteligível ^8^, de forma que a transgeneridade se torna invisível, o que garante um trânsito social mais seguro ^20^ ^,^ ^21^.

Partindo da realidade carioca de trans negros, representante da Liga Transmasculina João W. Nery complexifica a suposta facilidade deste grupo em obter emprego, uma vez que é profundamente marcada por violências, inclusive sexual. Sua reflexão, observando o Rio de Janeiro, sinaliza persistente imbricação de sexismo com racismo num sistema capitalista perverso, que desumaniza o corpo trans negro:

“*A maioria das pessoas está em espaços* [em] *que a educação não pode chegar, ou quando chega, ela é limitada. E os corpos dessas pessoas são colocados em outros lugares, ou para a prostituição, que é a objetificação da mulher preta, né? Porque é isso, a sociedade periférica não entende o corpo do homem trans preto, a sociedade branca, ela não entende isso, mas usa esse corpo para violentar ele de outra maneira e continuar escravizando. Então, a gente entende que é uma questão de classe também. É um outro trabalho, porque existe uma visibilidade que o homem trans, ele consegue emprego mais fácil, tudo isso, mas se a gente parar pra analisar, essa facilidade que é dita, vem com muita violência, com estupro, vem com objetificação, vem com silêncios, vem com o não falar, que antes já era imposto desde criança, por ser visto na sociedade como menina. Então, é algo da opressão das pessoas que tem vagina*” (Liga Transmasculina João W. Nery).

Estas ponderações sinalizam o quanto são necessários novos estudos a respeito da inserção social de pessoas transmasculinas. Se considerarmos, por exemplo, que a taxa de ocupação das mulheres na força de trabalho brasileira era de 52,7% frente a 72,3% dos homens no último trimestre de 2023 ^22^, é possível supor que homens trans também se beneficiem, em alguma medida, da misoginia presente na sociedade. Todavia, dadas as complexidades que envolvem as transmasculinidades e a ausência de dados oficiais sobre sua inserção no mercado de trabalho, fica aqui uma indagação a ser respondida futuramente.

#### Política de assistência social e segurança alimentar

Uma das medidas governamentais de enfrentamento à pandemia no âmbito da política de assistência social consistiu em auxílio financeiro, via transferência de renda, para famílias que necessitassem e para as inseridas no Cadastro Único, porta de entrada para acesso a benefícios sociais. O chamado Auxílio Emergencial foi motivo de polêmicas, do valor oferecido às dificuldades para solicitá-lo, seja pela ausência de recursos tecnológicos para uso do aplicativo CAIXA Tem pela população mais pobre, seja pelas filas imensas formadas nas agências da Caixa Econômica Federal, banco que centralizou a operação.

É inegável que este auxílio possibilitou o sustento de diversas famílias, ainda que de forma precária e abaixo do valor do salário mínimo. No entanto, muitas/os trans sequer o receberam: “*por conta, às vezes, de divergência entre o nome retificado e o cadastro antigo do CRAS* [Centro de Referência e Assistência Social]” (Liga Transmasculina João W. Nery). A representante do Instituto Trans da Maré conta que “*a gente teve que* (...) *pegar com duas pessoas o computador para ajudar algumas meninas a se inscreverem no auxílio*”.

Vale salientar que segurança alimentar e nutricional é a garantia do direito de todas as pessoas ao acesso a alimentos de qualidade, em quantidade suficiente e de modo permanente, com base em práticas alimentares saudáveis e respeitando as características culturais de cada povo, manifestadas no ato de se alimentar ^23^. Todavia, a pandemia agudizou a vulnerabilidade social, tendo a fome como sua maior expressão:

“*A ideia não era só vacinar, a ideia era alimentar, proteger, isolar. Por que como essas pessoas vão ficar isoladas? Não tem o que comer. Agora, dentro desse quesito da alimentação, as pessoas trans foram muito massacradas.* (...) *não teve um programa, certo? Um programa específico voltado para a população trans, como deveria ter tido*” (IBRAT).

Com isso, alguns coletivos desenvolveram ações específicas para pagamento de aluguel residencial, gás e mesmo alimentos. A criação de parcerias institucionais para doação de cestas básicas foi relatada por representante do IBRAT, por exemplo. Todavia, a solidariedade que marcou aquele período se dirigiu a apenas alguns:

“*Enfrentamos muito preconceito também, discriminação, por algumas ONGs e associações que estavam doando cestas básicas. Quando a gente falava que a gente era trans e travesti e que a gente precisava de uma cesta básica, eles não davam. Eles falavam que era mais pra família. Ah, você tinha que ter família pra comer?*” (Instituto Trans da Maré).

Chama a atenção a suposta oposição entre “família” e “transexualidade/travestilidade”, tão presente no discurso conservador brasileiro. Com o fortalecimento da extrema-direita, pânicos morais são acionados, visando promover resistência social a mudanças em hierarquias vigentes, como as de gênero e raça. Neste sentido, os movimentos feminista e LGBTI+ têm sido acusados de colocar em risco a instituição familiar, definida a partir de um ideal pautado pela cisheteronormatividade e pelo patriarcalismo ^24^. Este imaginário de perigo contribui para exclusão e desumanização de pessoas trans, a ponto de não receberem o alimento que necessitavam. O paradoxo está no fato de que a definição do que é reconhecido como vida, que vai mobilizar a sociedade a fim de mantê-la, re/produz o que deseja excluir:

“*A figura viva fora das normas da vida* [onde se situa a vida trans] *não somente se torna o problema com o qual a normatividade tem de lidar, mas parece ser aquilo que a normatividade está fadada a reproduzir: está vivo, mas não é uma vida. Situa-se fora do enquadramento fornecido pela norma, mas apenas como um duplo implacável, cuja ontologia não pode ser assegurada, mas cujo estatuto de ser vivo está aberto à apreensão*” ^25^ (p. 22).

#### Política de saúde e saúde mental

Um dos princípios do Sistema Único de Saúde (SUS) é a universalidade de acesso aos seus serviços, em todos os níveis de assistência, de forma igualitária, ou seja, sem preconceitos ou privilégios de qualquer espécie. Isto não se materializa, contudo, na experiência trans, como largamente apontado na literatura. O desrespeito ao nome social, o preconceito de profissionais e o diagnóstico patologizante no processo transexualizador são obstáculos ao cumprimento desse princípio ^25^ ^,^ ^26^. Os achados deste estudo aproximam-se desse diagnóstico. O desrespeito ao nome social e à identidade de gênero assumida permanece sendo um entrave:

“*Às vezes, tem recusa* [institucional] *de emitir um cartão com nome social ou, às vezes, trata no masculino, ou a ala no alojamento,* (...) *enfim, as demandas são diversificadas, mas elas sempre giram na questão da violação de direitos, né?!*” (GPV).

O agravamento deste quadro aconteceu quando, no início da pandemia, os atendimentos ambulatoriais foram suspensos por não serem considerados essenciais. O foco era organizar os hospitais para atender pessoas com COVID-19. Com isso, homens trans ficaram impossibilitados de comprar seus hormônios: “*Os medicamentos hormonais para mulheres trans, você ainda consegue comprar sem receita, mas dos homens trans você não consegue, acredito que para eles tenha* [sido] *um momento bem complicado*” (GPV). Todavia, a seriedade da situação é tal que para uma das pessoas entrevistadas, a interrupção do serviço não foi o problema mais grave:

“*O caso de saúde transmasculina no Brasil, no Rio de Janeiro, é muito defasado. O problema não foi os ambulatórios fecharem, o problema é muito maior, porque os nossos hormônios não podem chegar aqui se não for de forma ilegal. Então, o problema não foi só a pandemia, é algo que lá atrás já acontecia, porque se dá algum problema na fábrica, no lote, a gente fica sem hormônios por meses, é algo que vai acumulando. Então, quando chega pra gente, já chega atrasado. E, na verdade, a conta da saúde vai ficando mais cara porque se isso não é acompanhado, uma hora a bomba chega, sabe?*”.

Alto custo dos medicamentos, elevação dos preços e extensas filas para obtê-los gratuitamente pelo SUS foram desafios sinalizados:

“*O preço dos hormônios subiu 400 vezes, preço absurdo, né? O que custava R$ 30,00 e poucos reais, agora está custando R$ 240,00 ou R$ 250,00.* (...) *o Estado, por exemplo, ele consegue, ele oferta essa medicação gratuita em alguns ambulatórios, São Paulo, Rio, Brasília, esses que são os estados maiores. Mesmo assim, esse acesso à essa oferta é dificultoso, não é todo mundo, é uma fila muito grande para se ter essa medicação gratuita, porque ela tem esse custo muito alto. Por exemplo, uma ampola* [nome do fármaco ocultado] *custa de R$ 600,00 a R$ 800,00. Uma ampola, uma só! Que é um dos melhores hormônios hoje. E nem todo mundo tem esse dinheiro pra comprar uma ampola, né?*” (IBRAT).

As dificuldades denunciadas levam pessoas trans a realizarem hormonização por conta própria, o que pode incluir o uso de silicone industrial, trazendo inúmeros riscos à saúde. Até mesmo o uso prolongado de hormônios, sem o devido acompanhamento, pode resultar no desenvolvimento de doenças graves, inclusive câncer ^27^. Já a interrupção hormonal pode implicar no retorno de características corporais que estavam sendo bloqueadas pela medicação, tal como aborda representante da FONATRANS:

“*A questão da interrupção de alguns tratamentos, né? Principalmente a parte hormonal. E aí tem a questão das disforias, que dependendo do tempo de utilização dos hormônios, parando de tomar os hormônios, isso pode acarretar algumas coisas, as minas passarem a ter pêlos no corpo. Então, isso causa problemas internos pra elas, né? Os meninos voltando a menstruar, algumas coisas nesse sentido*. (...) *acaba impactando na parte de saúde mental*”.

Além de repercussões na saúde mental, há risco de homens trans/transmasculinos engravidarem, o que tenciona um sistema de saúde baseado na cisgeneridade. Não à toa, o Supremo Tribunal Federal instituiu o uso da categoria parturiente. Estudo recente aponta implicações negativas na experiência de pré-natal e parto de homens trans, dada a lógica cisheteronormativa presente na atenção à saúde e nos sistemas de informação, impossibilitando conhecermos a prevalência de gestações nesse grupo social. Entretanto, mapeamento realizado pelo IBRAT e publicado em 2023 apontou que 3,6% dos 900 homens transexuais ouvidos já gestaram. Em pesquisas perinatais, a linguagem cisheteronormativa invisibiliza a experiência desses parturientes ^28^. Consequentemente, necessidades específicas em saúde reprodutiva desta população não são re/conhecidas, em prol de se manter a fixidez de corpos e identidades. A plasticidade do corpo, enquanto passível de ser moldado por meio de tecnologias e práticas ^29^ é, entretanto, continuamente denunciada pela experiência trans, ao ponto de descolar o gênero da capacidade de gerar.

Na contramão dos apagamentos, até mesmo avanços obtidos transformam-se em barreiras:

“*Por exemplo, em 2018, o STF* [Supremo Tribunal Federal] *decretou o processo de retificação de nome.* (...) *Isso foi gerando outros problemas, por incrível que pareça, porque* (...) *quando eu ia fazer uso, por exemplo, do serviço ginecológico, eu era interrompido, porque o sistema SUS não entende um homem fazendo um atendimento ginecológico ou obstétrico*” (IBRAT).

Pertinente à denúncia de que “a conta vai ficando mais cara”, haja visto os riscos de homens trans/transmasculinos desenvolverem problemas ginecológicos, assim como o adoecimento urológico de mulheres trans, decorrentes das barreiras aos serviços. A organização do SUS possibilita que atendimentos considerados mais básicos sejam realizados em postos de saúde ou clínicas da família, nos quais se desenvolvem ações preventivas. Se a população trans não for acolhida, poderá desenvolver doenças mais avançadas a longo prazo, demandando atendimento em média e alta complexidade.

As experiências relatadas, vivenciadas diariamente, acarretam adoecimentos, inclusive de saúde mental, e se agravaram frente à crise sanitária, como aponta a Organização Mundial da Saúde (OMS). A prevalência global de ansiedade e depressão aumentou 25% no primeiro ano pandêmico, decorrente de medidas de isolamento social, combinadas com estressores como solidão, medo de contrair o vírus, sofrimento e morte de pessoas queridas, luto e preocupações financeiras ^30^.

Não à toa, representante da FONATRANS desabafou:

“*O principal desafio foi me manter bem para poder encarar essas questões de transfobia. No sentido de saúde mental. Porque tava tudo muito confuso e você não ter contato com outras pessoas* (...) *Isso já te deixa debilitado ali e essa questão de você* (...) *não saber o que vai acontecer, não saber como é que pega, não saber se vai morrer, se não vai morrer, o que que vai acontecer*”.

O cuidado relativo à saúde mental foi uma das maiores demandas recebidas pelos coletivos. Falta de atendimento nos ambulatórios trans e de acesso aos medicamentos necessários levaram muitos/as trans ao desespero, inclusive ao suicídio:

“*O que nós ouvíamos nos grupos era isso, era o desespero das pessoas, porque não tinha, os ambulatórios estavam fechados, as farmácias estavam fechadas, ou não faziam a entrega dessas medicações, ou então não tinha, então isso foi muito desesperador, muita gente regrediu* [nos efeitos do tratamento hormonal], *muita gente teve problemas de saúde em relação a isso e aí não é só saúde física, saúde mental. Não à toa houve o suicídio do* [nome ocultado], (...) *na carta dele, ele fala sobre isso,* (...) *que o acesso à saúde, o acesso a uma hormonização. E o suicídio dele foi no início da pandemia e então daí você tira o desespero, como foi muito grande. Desse caso você pode ampliar aí para muito mais*” (IBRAT).

O suicídio relatado foi de um jovem negro trans. Em entrevista, sua mãe revela que efeitos do racismo e da transfobia desencadearam uma depressão, que o levou à sua morte. Sensível às implicações sociais sobre o falecimento, o jornal optou por informar que o jovem havia sido “suicidado” ^31^. Uma das lideranças entrevistadas também fez este uso provocador para evidenciar o caráter suicida presente no desamparo do Estado, frente à necessidade de manutenção das terapias hormonais:

“*A gente teve um grupo muito grande de suicídio de homens trans na pandemia. Então, eles se fecharam mais, eles se deprimiram mais e*... *a falta de hormônio na farmácia fez com que eles sofressem, sofressem. É, eu digo, muitos meninos, além de uns tentarem e outros conseguirem ser suicidados*” (Liga Transmasculina João W. Nery).

Em 2020, registraram-se 23 casos de suicídio; em 2021, 12 casos; e em 2022, 20 casos. Provavelmente, há subnotificação desses casos. Por um lado, sendo o suicídio um tabu, muitas das mortes não são divulgadas como tal. Por outro, como diversas famílias e profissionais responsáveis pela notificação fúnebre não aceitam a transgeneridade, também não a respeitam no registro ^32^.

As razões que levam trans a cometeram suicídio perpassam por variadas situações de violências rotineiras, seja no ambiente familiar, seja no mercado de trabalho formal, por exemplo. Amiúde, estas são agravadas por outras opressões como o racismo, levando à desesperança, baixa autoestima, transtornos de ansiedade e depressão. Nesse sentido, vale reiterar o clamor da ANTRA para que suicídio e transfobia sejam tratados pelo viés da saúde pública, pautada em estudos e prevenção, de forma contínua e eficaz ^32^.

Os resultados apontados em suas várias dimensões tratam de pessoas cujos corpos a cisnormatividade não reconhece existência, como já dito, ou seja, são destituídas do privilégio ontológico. Neste caso, o domínio da ontologia é entendido como “território regulamentado” ^33^, no qual o que o constitui e o que dele é excluído são efeitos de relações de poder que o atravessam. Trata-se de uma produção diferenciada do humano e do abjeto (os excluídos), em que este aparece como figura socialmente indistinta. Logo, cabe destacar a relevância das reflexões que envolvem a constituição político-identitária trans e que aqui aparecem na diferenciação entre homens trans e transmasculinos. Como as construções discursivas se colam ao corpo dando vida a ele.

Ao propor a abjeção como processo, Butler ^16^ evita indicar quais são os corpos abjetos, talvez porque, a depender das relações em curso e da perspectiva de quem observa, a ontologia do ser possa ser reconhecida ou destituída. Neste estudo, dada a transfobia presente na sociedade brasileira, entendemos os corpos trans nesse lugar da abjeção. Isso explica, ao menos em parte, o descaso ou cegueira do Estado frente às necessidades específicas dessa população.

## Considerações finais

Os achados deste estudo sinalizam que os desafios impostos pela crise sanitária relacionada ao COVID-19 se combinaram a exclusões presentes na vida trans, seja pela transfobia institucional, que levou a ausência de iniciativas governamentais convergentes à realidade dessa população, seja pela transfobia manifesta nas relações cotidianas, que legitimou, por exemplo, a recusa de ajuda alimentar sob o argumento de que se destinava “apenas a famílias”. Os relatos denunciam dificuldade de acesso aos serviços de saúde, fome, adoecimento mental, maior exposição às situações de violência, tanto decorrente da necessidade de retorno ao ambiente familiar, no qual já sofriam discriminação, quanto de permanecerem trabalhando nas ruas, em um cenário em que até delegacias de polícia funcionaram em regime de plantão e/ou digitalmente. As violências relatadas foram tanto físicas como psicológicas e simbólicas, de estupro ao revés compulsório no processo de transição de gênero decorrente do fechamento inicial dos ambulatórios.

Apesar das dificuldades, observamos a importante atuação de coletivos que se organizaram para dar o suporte negligenciado pelo Estado. Dentre as iniciativas realizadas, foi possível identificar: doação de cestas básicas, fornecimento de insumos para higiene pessoal, atendimento profissional ou atividades virtuais direcionadas ao cuidado em saúde mental, distribuição de máscaras e álcool em gel, oferta de capacitações para geração de renda, suporte tecnológico e profissional para preenchimento do Cadastro Único, produção de dados de pesquisa e suporte jurídico. Assim, conseguiram desenvolver estratégias para suplantar o “cistema” e continuar existindo e resistindo por si e pelos outros, apesar de este reiteradamente insistir em sua exclusão. Revela-se aí a centralidade da luta política e da organização de coletivos nesse enfrentamento.

Ainda que o uso da internet tenha possibilitado o desenvolvimento de inúmeras pesquisas na pandemia, é necessário reconhecer suas limitações, particularmente, para realização de entrevistas, uma vez que a comunicação não-verbal se reduz ao enquadramento tecnológico. Apontamos aqui um primeiro limite deste estudo. Um segundo, refere-se ao número reduzido de entrevistas, que ainda assim nos trouxeram considerável riqueza de dados para análise.

Urge a adoção da perspectiva interseccional na elaboração de políticas públicas, especialmente aquelas voltadas à resposta a graves crises sociais, sejam elas sanitárias, climáticas ou econômicas. Ao permitir compreender como diferentes marcadores sociais (re)produzem desigualdades, a interseccionalidade se torna ferramenta profícua para desenho de estratégias que considerem vulnerabilidades específicas de cada grupo social, o que pode elevar suas chances de sucesso. Com isso, os resultados apresentados, voltados a um grupo tão estigmatizado socialmente e sobre o qual tem-se lacunas de conhecimento (como já apontado), contribuem com o esforço global, protagonizado pela Organização Mundial da Saúde, de preparação e resposta a futuras pandemias.

Recomenda-se também que políticas de respeito e valorização da diversidade sejam desenvolvidas, visando a inclusão social de grupos minorizados, como o de trans. Neste sentido, faz-se mister a ruptura com uma compreensão de “gênero” alicerçada na norma biológica para compreensão das transgeneridades como possibilidades de existências dignas de serem vividas.

## Data Availability

Os dados de pesquisa não estão disponíveis.
